# The Validity of Connecting Conversations: A Narrative Method to Assess Experienced Quality of Care in Nursing Homes from the Resident’s Perspective

**DOI:** 10.3390/ijerph17145100

**Published:** 2020-07-15

**Authors:** Katya Sion, Hilde Verbeek, Sil Aarts, Sandra Zwakhalen, Gaby Odekerken-Schröder, Jos Schols, Jan Hamers

**Affiliations:** 1 Department of Health Services Research, Care and Public Health Research Institute, Maastricht University, Duboisdomein 30, 6229 GT Maastricht, The Netherlands; h.verbeek@maastrichtuniversity.nl (H.V.); s.aarts@maastrichtuniversity.nl (S.A.); s.zwakhalen@maastrichtuniversity.nl (S.Z.); jos.schols@maastrichtuniversity.nl (J.S.); jph.hamers@maastrichtuniversity.nl (J.H.); 2 Living Lab in Ageing and Long-Term Care, Duboisdomein 30, 6229 GT Maastricht, The Netherlands; 3 Department of Marketing and Supply Chain Management, School of Business and Economics, Maastricht University, Tongersestraat 53, 6221 LM Maastricht, The Netherlands; g.odekerken@maastrichtuniversity.nl

**Keywords:** narrative, quality assessment, validity, interviews, relationship-centered care, quality of care, triad, resident perspective

## Abstract

It is important to assess experienced quality of care in nursing homes, as this portrays what is important to residents and helps identify what quality improvements should focus on. Connecting Conversations is a narrative method that assesses experienced quality of care from the resident’s perspective in nursing homes by having separate conversations with residents, family, and professional caregivers (triads) within a learning network. This study assessed the validity of performing the narrative method, Connecting Conversations. Trained nursing home staff (interviewers) performed the conversations in another nursing home than where they were employed. In total, 149 conversations were performed in 10 nursing homes. Findings show that experts deemed the narrative assessment method appropriate and complete to assess experienced quality of care (face validity). The questions asked appeared to capture the full construct of experienced quality of care (content validity). Additionally, there was a range in how positive conversations were and first results indicated that a nursing home scoring higher on satisfaction had more positive conversations (construct validity). More data are needed to perform additional construct validity analyses. In conclusion, Connecting Conversations shows promising results for its use as a valid narrative method to assess experienced quality of care.

## 1. Introduction

Worldwide, there is an increase in older people and, hence, an increasing demand for long-term care services, such as nursing home care [1,2]. Nursing homes are a type of long-term care service with 24–hour care and functional support for the most vulnerable people in our society with complex health needs [3]. The Institute of Medicine defined six domains to help define and assess quality of care: safety, effectiveness, efficiency, timeliness, patient-centeredness, and equitability [4]. It is challenging to assess quality of care, as providing care is a service that is characterized by its intangible, heterogeneous, multifaceted, perishable, and interactive characteristics [5,6]. Therefore, measures have been developed to assess a range of quality indicators, mostly focused on safety and effectiveness, such as the incidence of pressure ulcers [7]. As the data collected with quality measures are used for quality improvement, policy-making, accountability, and transparency, it is important to ensure that the quality indicators truly measure the construct they aim to measure [8,9,10]. 

Over the past decade, the nursing home culture has shifted from a mere medical approach to a more holistic, person- and relationship-centered approach, acknowledging the resident’s perspective, experiences, and caring relationships [11,12,13]. This holistic approach requires additional assessments of quality of care from the resident’s perspective, as amongst others, this can help care teams to improve quality, and it can support residents to enhance their quality of life in the nursing home [14,15]. Quality of care from the resident’s perspective is a process of care experiences with expectations before, care interactions during, and an assessment of the experience afterward in a certain context, as presented in the Individually Experienced Quality of Post-Acute and Long-Term Care (INDEXQUAL) framework [16]. Expectations are influenced by personal needs, previous experiences, and word-of-mouth [5]. The experiences in the caring environment are formed by the caring relationships among the resident, their family, and professional caregivers, and their interactions [17,18]. Therefore, it is important to include the professional caregivers’ and families’ perspectives as well when assessing quality of care from the resident’s perspective [19,20]. After the experience, an assessment is given of what happened and how it happened (perceived care services), how this impacted the resident’s health status (perceived care outcomes), and how this made the resident feel (satisfaction) [21,22]. 

Until now, the most common approach to assess residents’ quality of care has been with quantitative satisfaction, patient-reported experience, and patient-reported outcome measures, such as the Consumer Quality Index or the Net Promoter Score [7,22,23,24,25,26]. These measures however are not sufficient to capture quality of care from the resident’s perspective, as they only assess individual elements of care experiences and are lacking the meaning behind the response to these items [21,27]. To capture the full process of residents’ quality of care, it is valuable to use narratives, as these possess emotions, explain logic, provide information about the caring relationships, and capture an experience [28]. Narrative inquiry has been characterized by three dimensions: (1) personal and social (interaction); (2) past, present, and future (continuity); and (3) place (situation), and respondents receive the opportunity to share their stories and elaborate on points for improvement [29,30]. Therefore, narratives can help discover what is meaningful to residents and help to improve quality of care tailored to the individual [31]. Research has shown that care staff can use narratives to evaluate and improve care services based on care recipients’ stories [32].

The development of assessment methods is a stepwise approach in which the constructs and components are defined, the method is pilot- and field-tested, and reliability and validity are assessed [10]. Determining the reliability and validity of assessment methods is important to assure the quality of the method and the corresponding data, and to provide potential users transparency when selecting an appropriate assessment method [10]. Reliability and validity of narratives are usually assessed with four key components related to trustworthiness: credibility, transferability, dependability, and conformability, mainly focused on the process of data collection and analysis [33]. However, these components have been developed for qualitative research in general, not specifically for a qualitative assessment method [34]. 

Reliability is a prerequisite of validity and has been defined as “the degree to which measurement is free from measurement error” [35]. For qualitative assessment methods, the data are in a narrative form and subjective, and the interviewer is considered to be part of the method and can contribute to the reliability through training and practice [34,36,37]. Therefore, reliability of narrative methods in terms of consistency can be analyzed by evaluating the procedures of how the assessments are performed [38].

Validity has been defined as “the degree to which an instrument truly measures the construct(s) it purports to measure” [35]. It evaluates whether an assessment method actually measures a construct and whether the scores of the method are consistent with a theoretical framework of that construct [10]. The question is how validity of narrative assessment methods should be evaluated and whether the concepts of face, content, and construct validity can be used, as these have been developed to evaluate quantitative assessment methods [35]. Valid methods assessing quality of care contribute to the credibility of the quality-of-care data [39,40].

In the Netherlands, the use of narratives in nursing homes is occurring more frequently nowadays, as policy guidelines recommend the use of residents’ experiences for quality monitoring and improvement [41]. However, to date, little research has been done on the reliability and validity of these narratives and, if this has been addressed, this has usually been done by means of trustworthiness for qualitative research [10,42,43]. The data collected with these narrative quality assessment methods are being used in daily nursing home practice for quality improvements and policy-making, and therefore it is inevitable to determine their validity. 

Recently, the narrative method “Connecting Conversations” was developed aimed at assessing the entire process of experienced quality of care in nursing homes from the resident’s perspective [44] Connecting Conversations trains nursing home staff to perform separate conversations with a resident, family member, and a professional caregiver of that resident (triad). Its theoretical foundation is based on relationship-centered care and the full care experience as defined in the INDEXQUAL framework [16,45]. Connecting Conversations’ feasibility has been assessed by evaluating the consistency of the procedure in terms of performance completeness, protocol adherence, and satisfaction and has been published separately in this special issue of IJERPH as well [44]. This study aimed to evaluate the validity of performing the narrative method, Connecting Conversations.

## 2. Materials and Methods

The study used a mixed-methods cross-sectional design and data collection was performed from October 2018 to February 2019.

### 2.1. Connecting Conversations 

Connecting Conversations is a narrative method that assesses experienced quality of care in nursing homes from the resident’s perspective. Separate conversations are performed with the resident, a family member, and a caregiver involved in the daily care of that resident (a triad) by a nursing home staff member (interviewer) employed in another care organization than where he or she performs the conversations. This provides for a learning network, creating the opportunity for interviewers to learn from each other and another environment, and it enhances an equal relationship between the participants in the triad and the interviewer. The method is based on appreciative inquiry, which focuses on what is going well and how this can be done more, instead of only focusing on problems and the negative [45].

The six main Connecting Conversations questions are about the resident’s life; satisfaction with care provision; most positive experience; description of an average day in the nursing home; and relationships between the resident, family, and caregiver, based on the INDEXQUAL framework [16]. Interviewers received simple visuals (green, yellow, and red smiley) to support residents in answering the questions when needed. To ensure that interviewers have all the knowledge and skills to perform the conversations, a 3-day training is provided by UMIO, an executive branch of the university, in which interviewers learn to perform the conversations. During day 1 and 2 interviewers are taught that the questions in the protocol should be used to trigger respondents to share their stories and can be supported with conversation techniques, such as responding with probing questions, paraphrasing, and creating purposeful silences. Day 3 is focused on sharing experiences, reflecting, and learning with and from each other. Specific details on the narrative method have been published separately in this special issue of IJERPH [44].

### 2.2. Interpretation and Operationalization of Validity for Connecting Conversations

In total, three concepts were assessed for Connecting Conversations: (1) face validity, (2) content validity, and (3) construct validity [10]. Table 1 presents the definitions of these concepts for a narrative method, the operationalization of these concepts for “Connecting Conversations” and how they were translated into an analysis [35].

### 2.3. Setting and Participants

Care triads and interviewers were recruited from the nursing homes within the Living Lab in Ageing and Long-Term Care South-Limburg [46]. 

#### 2.3.1. Care Triads

In the Netherlands, there are different types of nursing home wards that offer long-term somatic care for residents with physical disabilities, long-term psychogeriatric care for residents with dementia, or temporary rehabilitation care [47]. This study included triads with residents living in both somatic and psychogeriatric wards. Ten nursing homes each selected one ward if 15 or more residents lived in a ward or two wards if less than 15 residents lived in a ward. 

Within each ward, five triads (wards <15 residents) or ten triads (wards >15 residents) were recruited randomly by the research team in collaboration with a contact person of the ward. Random selection aimed to avoid selection bias and ensured a true sample of residents’ experiences on the ward could be captured. One triad consisted of a nursing home resident, a family member, and a caregiver of that resident. Inclusion criteria were that the resident was living in the nursing home and received long-term care at the time of the conversation; the family member was the nursing homes’ first contact person for the resident; and the caregiver was involved in the residents’ daily care provision at least one day a week. 

Random selection of triads was performed by generating a random sequence list of all residents’ room numbers in a specific ward. The contact person of the ward asked residents of the first 5 (or 10) randomized room numbers if they were interested in participating. When a resident refused, the next was approached until 5 (or 10) residents (and thereby, triads) were recruited. The reasons for randomizing all room numbers, prior to asking whether participants would be interested to join were threefold. First, this assured all residents received an equal chance of being included for the conversations. There is risk of selection bias when recruiting residents for conversations, as well-spoken, more involved residents and families are more likely to respond to the recruitment call. This occurred during pilot testing of the narrative method. By randomizing all resident room numbers, each has an equal chance of being selected and invited to participate. Second, the opportunity to give the resident a voice was not limited by the willingness of the family member to participate. Third, once a participant has been randomly selected and is willing to participate, he or she will have the certainty that this will happen. This avoids getting their hopes up and eventually them not being selected for the conversations. Only once a resident agreed to participate, the family and professional caregiver were approached. If the resident was unable to have the Connecting Conversations because of cognitive impairment, the triad was included as a dyad (family–professional caregiver). If no family member was available or the family did not want to participate, the triad was also included as a dyad (resident–professional caregiver). If a professional caregiver did not want to participate, he or she recommended another caregiver closely involved in the resident’s care to participate.

#### 2.3.2. Interviewers

Any staff member interested in becoming an interviewer could apply, and managers selected interviewers based on their intrinsic motivation and involvement in quality assurance by providing hands-on care or within a policy position. Additionally, a health scientist and psychologist employed at the university attended the training and performed conversations as well. Selection aimed at including 12 to 20 interviewers, as this was a suitable group size for participation in the intensive, highly interactive training.

### 2.4. Data Collection and Procedure

#### 2.4.1. Procedure

Interviewers’ demographic characteristics were collected at the start of training day 1. These were age in years, sex, job title, and years of working experience in the nursing home setting. The research team assigned interviewers to another nursing home than where they were employed to perform Connecting Conversations. Each interviewer was instructed to perform conversations with five full triads on a ward. Interviewers scheduled their own conversations with a contact person in their assigned nursing homes. They could perform multiple one-hour conversations a day. Family members who were unable to attend a face-to-face conversation were interviewed by phone. Interviewers audio recorded and documented a summary per question on a tablet. 

#### 2.4.2. Face Validity

Key stakeholders, client representatives, and interviewers were invited to express to what degree they judged Connecting Conversations to be an appropriate method to assess experienced quality of care in nursing homes. Key stakeholders (up to two per institution) were from the Dutch Ministry of Health, the Dutch Health Care Institute, the Dutch Client Council, the Dutch Professional Association of Nurses, the Dutch Health and Youth Care Inspectorate, and the board members of Nursing Homes. Up to three client representatives per care organization were invited through the seven care organizations within the Living-Lab of Aging and Long-Term Care [46]. 

Two separate interactive group discussions were scheduled, one for key stakeholders and one for client representatives, which were documented in meeting minutes. Participants discussed two questions: (1) To what extent do you judge Connecting Conversations to be an appropriate method to assess quality of care in nursing homes from the resident’s perspective? and (2) To what extent do you judge the questions asked with Connecting Conversations to fully cover the concept of experienced quality of care in nursing homes from the resident’s perspective? Interviewers evaluated all three training days and field notes were taken. First, information on the background and development of Connecting Conversations was presented. Thereafter, participants were invited to express their thoughts on the design of Connecting Conversations and provide the research team with constructive feedback. 

#### 2.4.3. Content Validity

To assess the degree to which Connecting Conversations has a sample of questions that covers the full concept of residents’ experienced quality of care as defined by the INDEXQUAL framework, separate conversations with resident–family–caregiver triads were performed and audio-recorded, according to the Connecting Conversations protocol.

#### 2.4.4. Construct Validity

In the Dutch national quality framework for nursing homes, the Net Promoter Score (NPS) is currently the minimally required assessment for residents’ experiences in nursing homes [41]. Therefore, all participating nursing homes were offered the choice of whether they wanted the NPS to be measured in their nursing homes alongside Connecting Conversations. The NPS is a one-item measure that assesses loyalty, as a derivate for satisfaction, by asking residents one question: “on a scale of 0–10, would you recommend this nursing home to your family and friends?” A score of 9 or 10 is a promoter, and scores of 6 or below are detractors. The final NPS score is a % calculated as the different between the % of promoters and the % of detractors [26]. In general, a more positive score (>0) is considered good and a more negative score (<0) is considered poor. The NPS was considered a suitable comparator to validate Connecting Conversations’ data, as it also assesses the more subjective side of quality of care from the resident’s perspective. It differs from Connecting Conversations as it only provides a basic one-score rating, without reaching the underlying explanation of why this score has been given.

### 2.5. Data Analysis

#### 2.5.1. Face Validity

Field notes and meeting minutes were formatted and analyzed by the first author. Data were categorized into two components: appropriateness and completeness. Within appropriateness, feedback on the appropriateness of the method was extracted, such as opinions on the choice for a narrative form or the three separate conversations. Within completeness, feedback on the number and content of questions was extracted, such as the formulation of the questions or missing topics. Two researchers evaluated the comments during two face-to-face discussions during which the categorized findings were interpreted. 

#### 2.5.2. Content Validity

A sample of all collected data was selected for validity analysis to avoid overrepresentation of an interviewer or ward. One completed triad per interviewer, which was audio recorded, was randomly selected. The random sample of transcripts was coded with the 15 themes from the INDEXQUAL framework, as this framework covers the themes of experienced quality of long-term care. Directed content analysis was performed [48]. Both researchers independently coded the transcripts with the sub-themes from the INDEXUQAL framework [16]. Coding was supported with a code tree that defined each INDEXQUAL theme (Table 2). The INDEXQUAL framework consists of four main themes divided into 15 sub-themes. For each sub-theme, a question was formulated that enhanced the coders understanding of the code tree. If a section was unrelated to the INDEXQUAL sub-themes, it was left un-coded. Discrepancies between both researchers regarding the assignment of a code were discussed with the research team until consensus was reached. 

#### 2.5.3. Construct Validity

On a scale of 1 (bad) to 10 (perfect), responders are known to give a range of answers between 1 and 10. When using narratives, the range in answers provided is less standardized. Therefore, transcripts were coded with two codes: positive and negative, by two researchers independently. Segments were only coded if a clear emotional value was provided, for example positive segments included words such as “satisfied,” “happy,” “great” and negative ones such as “unfortunate,” “frustrating,” “angry.” Neutral segments such as “she reads a lot” were not coded. Per transcript, the total number of positive coded segments was calculated as a percentage of the total number of coded segments: e.g., if 50 segments were coded, of which 30 were positive and 20 were negative, the %-positive would be 60%. For each triad, the %-positive was plotted into a graph to visualize the range in %-positive between the different conversations (resident–family–caregiver) and different triads.

Additionally, the %-positive of triads performed in a participating nursing home with a high NPS (>0) in 2018, and a nursing home with a low NPS (<0) in 2018 were compared. Both NPS scores were compared to the nursing homes’ %-positive. Validity was apparent if the %-positive was lower in the nursing home with the lower NPS score compared to the %-positive of the nursing home with the high NPS score. This analysis was performed on all full triads available for both nursing homes. Qualitative data were analyzed with MAXQDA version 18.1.1. (VERBI Software, Berlin, Germany) and quantitative descriptive data with SPSS version 25 (IBM Nederland B.V, Amsterdam, The Netherlands) [49,50].

### 2.6. Ethical Considerations

The study protocol was approved by the medical ethics committee of the regional medical center Zuyderland (17-N-86). Information about the aim of the study, the expected burden of the conversations, and confidentiality was provided to all residents, family members, and caregivers in the triads in advance by letter. Before the start of each conversation, written informed consent was provided by all participants. Residents with legal representatives gave informed assent themselves before and during the conversations, and their legal representatives gave written informed consent [51]. Participation was strictly voluntarily and participants were allowed to withdraw from the study at any moment. In order to guarantee privacy and anonymity of participants, no names or organizations were documented. 

## 3. Results

In 2018, 16 interviewers attended the training and performed 149 Connecting Conversations (46 residents, 46 family members, 57 caregivers) in 10 different nursing homes (4 psychogeriatric, 5 somatic, 1 acquired brain injury <65 years). In total, 34 full triads were performed, 11 family–caregiver dyads, and 11 resident–caregiver dyads. Of these conversations, 125 were successfully audio recorded and 21 were not due to technical failure (n = 17), or participants refusal to audio record the conversation (n = 4). All interviewers attended the first two training days and 13 (81%) attended the third evaluation training day. Interviewers’ demographics are presented in Table 3.

Interviewers had planned to perform five completed triads each; however, multiple triads were not completed. Reasons for an incomplete triad included: cognitive inability of the resident to participate in the conversation (n = 11), unavailability of a family member to participate (n = 11), and challenges recruiting triads within a ward due to scheduling issues and lack of time (n = 23 triads). Table 4 presents a summary of the main findings for the validity analyses.

### 3.1. Face Validity

Key stakeholders (n = 7), interviewers (n = 16), and client representatives (n = 10) evaluated whether the design of and questions asked with Connecting Conversations were fitting to assess experienced quality of care in nursing homes from the resident’s perspective. All expressed the importance of taking time to perform conversations and the benefit of having three separate conversations. Additionally, key stakeholders highlighted the strength of the method being based on the INDEXQUAL framework: “it is important to include the resident’s experiences, but also the families’ and caregivers’ experiences” and client representatives confirmed, “to a large extent, the relationship with a resident determines the experienced quality of care.” Interviewers were able to reflect on the questions after having performed conversations and evaluated that “they are the correct questions to ask and very clear.” The main concern of key stakeholders and interviewers was whether residents with cognitive impairment would be capable to have these conversations; client representatives however did not express this concern. Interviewers, for example, suggested it would be good to “receive some more guidance and supportive tools.”

### 3.2. Content Validity

Of the 16 interviewers, 11 completed at least one full triad with audio recordings. The 11 triads were performed in somatic wards for older people (n = 5), psychogeriatric wards for older people (n = 5), and an acquired brain injury ward for people <65 years old (n = 1). 

Table 5 presents how often each INDEXQUAL sub-theme was coded with the INDEXQUAL framework. The larger the grey circle, the higher the number of coded segments. Additionally, Table 5 presents quotes for each sub-theme to enhance understanding of how the data fit the framework. Analysis showed that all themes and almost all sub-themes from the INDEXQUAL framework were present in the random selection of triads. These findings suggest that the six Connecting Conversations questions cover the full concept of experienced quality of care. Word-of-mouth is the only sub-theme that rarely occurs. Residents did not address the relationship between their family and professional caregivers, which makes sense, as they are not directly asked about this. Perceived care services, perceived care outcomes, and satisfaction were identified the most; in line with the INDEXQUAL framework that places these themes in the after “assessment” phase. Numerically, less segments were coded for residents (n = 404) compared to those for family members (n = 636) and caregivers (n = 621). 

### 3.3. Construct Validity

For each transcript within a triad, both positive and negative segments could be identified and coded. An example of a positive and a negative segment are presented below.

- Positive segment Resident-Caregiver (triad 008) - Interviewer: “How is the contact between you and Mister Johnson?” Caregiver: “Actually, it is very good. I experience it as being pleasant. He is very grateful that I am there for him and help him.”
- Negative segment Care environment (triad 002) - Interviewer: “Is there anything that could be better?” Resident: “Yes, the care provision. They are busy. They see everything but yeah… And the music is loud. I cannot stand that. Then I often ask if it can be softer.”

**Figure 1:**
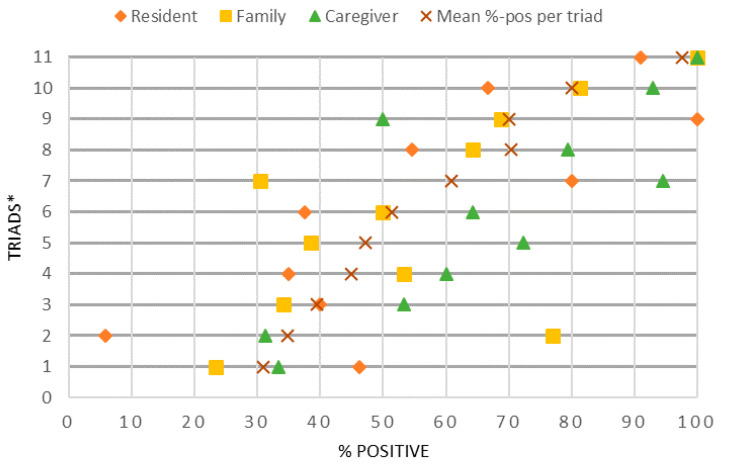
%-Positive coded segments of each resident, family, and caregiver per triad. * Each row represents one completed Connecting Conversation triad, presenting the %-positive for the resident, family, caregiver, and the mean %-positive for these three.

Figure 1 presents the range in quality ratings between conversations and triads. Each row represents a different triad and portrays the %-positively coded segments of the resident, family, and caregiver in that triad and the “x” shows each triad’s mean %-positive. For residents, %-positive ranged from 6% to 100%, for family it ranged from 23% to 100%, and for caregivers it ranged from 31% to 100%. These findings indicate that Connecting Conversations’ data capture a large variety in scores range from low %-positive to high %-positive. The median %-positive over the 11 triads is 54% and caregivers (64%) seemed more positive than residents (46%) and family members (53%).

We compared %-positives to the NPS-score for two nursing homes (Table 6). Nursing home A scored high above average on the NPS score (34) and shows that this nursing home scored a higher %-positive coded segment (72%). Nursing home B scored greatly below average on the NPS score (−50) accompanied with a lower %-positive (57%). This indicates that there is a convergence between resident satisfaction measured on a one-item scale (NPS) and the qualitative data (%-positive) collected with Connecting Conversations. There were insufficient data to perform a correlation analysis.

## 4. Discussion

This study assessed the validity of performing the narrative method “Connecting Conversations,” which aims to assess experienced quality of care in nursing homes by performing separate conversations with a resident, family, and professional caregiver of that resident. Results indicated that Connecting Conversations is a promising method to assess experienced quality of care in nursing homes from the resident’s perspective and appears valid. Experts reported that both the design and questions asked were deemed appropriate and complete to assess experienced quality of care (face validity). Thematic content analysis showed the full construct of experienced quality of care appeared to be captured with the conversations (content validity). When addressing construct validity, a range from negative to positive conversations became apparent. In addition, first results indicated a nursing home scoring low on satisfaction also scored a lower %-positive coded segments compared to a nursing home scoring high on satisfaction (construct validity).

Our findings show that narratives can be used to evaluate care services, confirming the conclusion from another study [32]. In nursing research, narratives are usually used to collect stories about someone’s experiences in a certain context [52]. However, stories collected with Connecting Conversations provided information on the full construct of experienced quality of care attached with a judgement of that quality, operationalized as %-positive. Quality of care is a complex concept and therefore it is recommended to assess multiple components including resident experiences, clinical outcomes, and employee satisfaction; for example, experienced quality of care assessed with Connecting Conversations, accompanied with the quantitative standardized quality indicators assessed with the National Prevalence Measurement of Quality of Care and employee satisfaction assessed with the single-item measure for overall job satisfaction [53,54,55]. By combining quantitative and qualitative data, we are able to capture a holistic view on quality of care [6,54]. This can contribute to more tailored policy-making and quality improvement at nursing homes’ operational (care triads), tactic (care teams), and strategic (care organization) levels, aimed at achieving higher quality of care within a nursing home [56]. 

Findings show residents living in nursing homes themselves are often capable of having conversations about their experienced quality of care, even when verbally challenged. The interpretation of stories shared by residents with moderate to severe cognitive impairment does need to be done cautiously. Research has shown this may be less valid, as residents may have difficulties correctly understanding questions and remembering past experiences [57]. Connecting Conversations strengthened this by having three separate conversations, i.e., by including the families and caregivers stories as well, known as data triangulation [33]. Findings show the benefit of including all three perspectives, as the %-positive between actors in a triad often differed. Additionally, research has confirmed that with trained interviewers and clearly formulated questions residents with cognitive impairment can more often be included in the conversations [14,58,59,60]. The interviewer may need to be provided with more support when conducting the conversations with the most vulnerable residents by means of more supportive questions and visuals, or by performing additional observations [61,62,63]. 

For this study, several methodological considerations need to be addressed and some suggestions for future research. First, coding %-positive was binary (positive or negative). In practice, this range is larger as “I am extremely happy” is interpreted as fully positive compared to “I am quite happy,” which is still positive, but to a lesser extent. We made no distinction between both types of positive quotes. Future research should focus on more in-depth analysis of the different intensities of positive and negative wordings, by means of for example text-mining [64,65]. This can contribute to an even better understanding of the similarities and differences between experienced quality of care according to residents, their families, and professional caregivers. Second, validity can only be present if an assessment method is reliable [66]. For quantitative assessment methods, reliability analyses are usually focused on the outcome of the method in terms of consistency, stability, and repeatability [10]. Future research should explore possibilities to assess reliability of the outcome for narrative methods by means of for example inter-rater reliability or test–retest [10]. Third, there were insufficient data to perform a correlation analysis with satisfaction outcomes. Additional assessments should be performed to analyze this and other types of construct validity, such as the known-groups method, to explore whether the method can distinguish nursing homes that are doing well compared to nursing homes that require more quality improvements [10]. This is challenging as there is no standard evaluation available for narrative methods and existing evaluations will need to be adapted.

The current study introduced a different approach than trustworthiness to evaluate the validity of a narrative method that assesses quality of care with face, content, and construct validity measures. It can be used by other researchers as a starting point to further explore validation of narrative assessment methods and can help to select appropriate qualitative methods that assess quality of care. When using the current study as an example, several steps should be taken into consideration. First, it is important to a priori clearly define the construct to assess, as analysis on validity focuses on this. Second, a selection should be made of which concepts of validity will be assessed and how these will be assessed. Third, these concepts should be clearly defined and operationalized to the narrative method under study, as transparency supports the thoroughness of the research [67,68]. 

## 5. Conclusions

The narrative method Connecting Conversations is deemed a promising method to assess experienced quality of care in nursing homes from the resident’s perspective. Using validated narrative methods can contribute to credible quality assessments that can help determine what is going well and what needs to be improved when delivering care. It is important to use validated quality assessment methods, as the accuracy of the collected data is a first step toward more effective quality improvement initiatives and policy-making. Therefore, it would be beneficial to standardize the reliability and validity analysis of qualitative assessment methods. For Connecting Conversations, research should collaborate with practice and policy to explore how to embed the narrative assessment method in practice and how the data can be used to improve experienced quality of care in nursing homes.

## Figures and Tables

**Table 1:** Validity definitions, operationalization, and analyses for Connecting Conversations.

Concept	Definition	Operationalization for Connecting Conversations	Analysis
1. Face validity	The degree to which a narrative assessment method looks as though it is an adequate reflection of the construct to be measured [35]	The degree to which experts, interviewers and client representatives judged Connecting Conversations actually assesses residents’ experienced quality of care in nursing homes	Three separate group discussions in which evaluations by key stakeholders, client representatives, and trained interviewers were interpreted
2. Content validity	The degree to which a narrative assessment method adequately represents the construct under study [35]	The degree to which Connecting Conversations has a sample of questions that covers the full concept of residents’ experienced quality of care as defined by the INDEXQUAL framework	Analyzed whether transcripts could be coded with the themes from the INDEXQUAL framework of experienced quality of long-term care for one full triad per interviewer
3. Construct validity	The degree to which the stories of a narrative assessment method are consistent with hypotheses, e.g., with regard to internal relationships, relationships with scores of other assessment methods, or differences between relevant groups [35]	The degree to which data collected with Connecting Conversations can be interpreted as ratings of experienced quality of care, varying from negative to positive	Analyzed the %-positively coded segments per transcript for one full triad per interviewer. Hereafter, compared %-positive to the actors within a triad and between triads
		The degree to which results from Connecting Conversations are similar to results from the Net Promoter Score (NPS), assessing residents’ loyalty/satisfaction	The %-positive coded segments were compared to the NPS score for all full triads of one nursing home scoring high and one scoring low on the NPS score

**Table 2:** Code tree Individually Experienced Quality of Long-Term Care (INDEXQUAL).

Theme	Sub-Theme	Interpretation
Context	Nursing home	What are the characteristics of the nursing home?
	Person	Who was and who is the resident?
Expectations	Expectations	What did the R–F–C expect from the nursing home care?
	Word-of-mouth	What did the R–F–C hear from others about nursing home care?
	Personal needs	What needs does the resident have? (sense of security, belonging, continuity, purpose, achievement, significance)
	Past experiences	What prior experiences did the R–F–C have with care?
Experiences	Experiences (daily routine)	What does an average day of the resident look like?
	Relationship-centered care	How are the relationships in the nursing home? (more general than themes below)
	● Resident–Family	How is the relationship between R–F?
	● Resident–Caregiver	How is the relationship between R–C?
	● Family–Caregiver	How is the relationship between F–C?
	Care environment	How is the subjective nursing home environment experienced?
Experienced quality of care	Perceived care services	What happened during a specific experience?
	Perceived care outcomes	How is the resident’s health status?
	Satisfaction	How did it make the R–F–C feel?

R: resident, F: family, C: caregiver.

**Table 3:** Interviewer demographics and data collection.

Interviewers (N = 16)	
Mean age in years (SD)	40 (11)
% Female	14 (88)
Occupation	
Nurse (%)	10 (63)
Policy advisor (%)	3 (19)
Nurse aid (%)	1 (6)
Psychologist (%) ^1^	1 (6)
Health scientist (%) ^1^	1 (6)
Mean contracted hours per week (SD)	32.3 (5.2)
Mean years working experience (SD)	13.8 (9.7)

^1^ Not employed in the nursing home, but at the university.

**Table 4:** Main findings face, content, and construct validity.

Concept	Interpretation Connecting Conversations	Main findings
1. Face validity	The degree to which experts, interviewers, and client representatives judged Connecting Conversations truly assesses residents’ experienced quality of care in nursing homes	Key stakeholders (n = 7), interviewers (n = 16), and client representatives (n = 10) evaluated the design of and questions asked with Connecting Conversations to be the right formula to assess experienced quality of care in nursing homes from the resident’s perspective
2. Content validity	The degree to which Connecting Conversations has an appropriate sample of questions to cover the full concept of residents’ experienced quality of care as defined by the INDEXQUAL framework	All themes and sub-themes from the INDEXQUAL framework were present in the 11 randomly selected triads. Word-of-mouth was seldom identified
3. Construct validity	The degree to which data collected with Connecting Conversations can be interpreted as true ratings of experienced quality of care. Thus, there is a variety in conversations from being not positive to very positive	%-positive ranged between and within triads ● Residents, 6% * to 100% positive ● Family, 23% to 100% positive ● Caregivers, 31% to 100%
	The degree to which results from Connecting Conversations are similar to results from the Net Promoter Score (NPS), assessing residents’ loyalty/satisfaction	A nursing home scoring low on the NPS also scored a lower %-positive compared to a nursing home scoring high on the NPS, showing a general tendency There was insufficient data for a correlation analysis

* 6% positive means 94% negative coded segments.

**Table 5:** Connecting Conversations content validity coded with INDEXQUAL themes.

Theme	Sub-Theme	R	F	C	Quote
Context	Nursing home	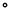	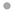	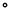	“It is eventually small-scale living.” (F)
	Person	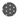	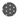	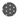	“She always enjoys to talk.” (C)“I am used to speaking dialect and that is what I feel comfortable with.” (R)
Expectations	Expectations	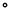	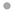	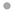	“What is being organized here, I have been totally amazed. I did not expect that.” (F)
	Word-of-mouth		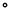		“Her husband also has that. They all think it is too busy.” (F)
	Personal need	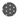	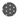	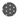	“But, close by, that is precisely what I long for. That I really live in my own village. And that is very important to me.” (R)
	Past experiences	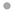	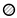	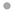	“I also think through the years, she used to live elsewhere. The family therefore has certain expectations of care that cannot always be achieved.” (C)
Experiences	Experiences (daily routine)	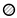	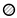	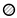	“In the evening she usually goes to bed on time, because she has dialysis and then she has to be downstairs at 7.30 a.m.” (F)
	Relationship-centered care	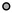	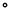	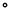	“The contact with the people from the other neighborhood here…she really misses that connection.” (C)
	Resident–Family	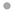	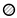	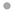	“It’s nice every time they visit.” (R)
	Resident–Caregiver	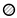	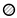	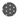	“She likes all staff, so a 10.” (F)
	Family–Caregiver		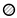	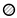	“Yes, actually good too; the daughter is also the first contact person.” (C)
	Care environment	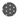	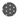	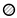	“Because, they don’t always have time for us.” (R)
Experienced quality of care	Perceived care services	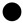	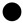	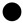	“Yes, you are looked after, but that is all. You have to nag the entire week because you don’t have absorbent products and then suddenly there are six packs on the rack.” (R)
	Perceived care outcomes	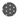	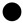	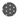	“She always used to love to read, but reading is not possible anymore.” (F)
	Satisfaction	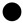	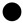	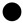	“Sometimes a bit annoyed.” (C)

C: caregiver, F: family, R: resident. The larger the colored circle, the higher the number of coded segments (calculated based on 20 percentiles). 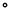 1–7 | 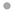 8–26 | 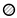 27–37 | 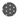 38–62 | 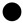 63–150 coded segments.

**Table 6:** NPS score and Connecting Conversations %-positive.

Measure	Nursing Home A		Nursing Home B	
	Score	*n*	Score	*n*
NPS score (residents)	34	38	−50	16
% Positive Connecting Conversations (residents)	62%	4	49%	3
% Positive Connecting Conversations (triads R–F–C)	72%	12	57%	9
